# Supplementary material for: Gait asymmetry, and bilateral coordination of gait during a six-minute walk test in persons with multiple sclerosis
Source: Sci Rep. 2020 Jul 24;10:12382. doi: 10.1038/s41598-020-68263-0 (PMC7382471; doi:10.1038/s41598-020-68263-0)
Supplement: Supplementary file 1 — Supplementary Information. [file 41598_2020_68263_MOESM1_ESM.pdf]

Supplementary material for:
Gait asymmetry, and bilateral coordination of gait during a six-minute walk test in persons with multiple sclerosis

Authors: Meir Plotnik*[123], Joanne M. Wagner PT, PhD[4], Gautam Adusumilli[5], Amihai Gottlieb[1], Robert T. Naismith, MD[5]

1 Center of Advanced Technologies in Rehabilitation, Sheba Medical Center, Ramat Gan, Israel.

2 Department of Physiology and Pharmacology, Sackler Faculty of Medicine, Tel-Aviv University, Tel Aviv, Israel

3 Sagol School of Neuroscience, Tel Aviv University, Tel Aviv, Israel

4 Department of Physical Therapy and Athletic Training, Saint Louis University

5 Department of Neurology, Washington University in St. Louis

*Corresponding Author: Meir Plotnik, PhD, Rehabilitation Hospital, Chaim Sheba Medical Center, Ramat Gan, 5265601, Israel.

Tel: +972-3-5307508

Fax: +972-3-5303821

Email: meir.plontik@sheba.health.gov.il

Supplementary material for:
Gait asymmetry, and bilateral coordination of gait during a six-minute walk test in persons with multiple sclerosis

Supporting material for Secondory analysis on the effects of gait speed

In the *Results* section we examplified how Pearson correlaiton coeficients (r_p) values were calculated (Fig. 3, body of article) to evaluate the interisnc (within subject) relation between the exmined variables (Stride CV, PCI and GA) and gait speed, as expressed by the distanced covered. This was done for each subject for each variable based on six data points, calcuaed from the measured perfomrance durign each minute of the six minute walk test (6MWT).

Values of r_p were used to conrol for the intrisic association between gait speed and each of the individual gait parameters (gait asymmetry (GA), stride CV and phase coordination index (PCI)) in order to study group effects on each gait parmeters sampeld during the 6MWT. To test whether there are group differences between the correlations (r_p values) between GA, PCI and CV and between distance covered (i.e., gait speed) among the three study cohorts, three ANOVA analyses were performed treating the correlations as a dependant factor and group as an independant factor. None of these analyses yielded a significant group effect, GA - $F(2,89) = 1.9$, $p=.15$, PCI - $F(2,89) = 0.3$, $p=.70$, CV - $F(2,88) = 1.0$, $p=.34$.

Herein we provide further infomration about the values of r_p seen for GA, stride CV and PCI.

For GA vs. distance covered within subject correlation, the mean value of r_p (± SD) was 0.01 ± 0.54 (ragne: -0.97 – 0.91). Forty five negative values were obtained, showing reduction in GA values (more symmetry) as distacne covered (i.e., gait speed) increases, and 47 positive values, showing the oposite trend, were obationed. Binomial test suggests that this proportion division is not significant ($p=0.458$). Out of the 45 negative trends, seven were statistically significant ($r_p \leq -0.822$; $p \leq 0.045$). Out of the 47 positve trends, five were statisically sginficant ($r_p \geq 0.829$; $p \leq 0.041$).

For stride CV vs. distance covered within subject correlation, the mean value of r_p (± SD) was -0.01 ± 0.50 (ragne: -0.90 – 0.90). Forty nine negative values were obtained, showing reduction in stride CV values (greater gait rhythmicity) as distacne covered (i.e., gait speed) increases, and 43 positive values, showing the oposite trend, were obationed. Binomial test suggests that this proportion division is not significant ($p=0.301$). Out of the 49 negative trends, five were statistically significant ($r_p \leq -0.842$; $p \leq 0.035$). Out of the 43 positve trends, five were statisically sginficant ($r_p \geq -0.853$; $p \leq 0.031$).

For PCI vs. distance covered within subject correlation, the mean value of r_p (± SD) was -0.17 ± 0.47 (ragne: -0.93 – 0.84). 61 negative values were obtained, showing reduction in PCI values (better coordination) as distacne covered (i.e., gait speed) increases, and 31 positive values, showing the oposite trend, were obationed. Binomial test suggests that this proportion division is statistically significat ($p=.0012$). Out of the 61 negative trends 10 were statistically significant ($r_p \leq -0.821$; $p \leq 0.045$), but only one positve trend was statistically significan ($r_p = 0.841$; $p = 0.036$).

Since each individual may have different values range (compare green triangles with red circles in figure 3 in the body of the article), and in order to combine cross subejcts data we transformed data points to Z-scores values according to the formula:

$$(1)\ Z = (x-\mu)/\sigma$$

where x is the data poitn value (six data points), and μ and σ are the mean and the standard deviation for the parameter (calculated for each subject separately).

The transfomration was perefomed separtely for each paticipant, for the distance covered and for stride CV, GA and PCI based on each individual's μ and σ values. Figure S-1 depicts the combined data set of PCI

Supplementary material for:
Gait asymmetry, and bilateral coordination of gait during a six-minute walk test in persons with multiple sclerosis

Z-scores values plotted agains distance covered Z-scores values. Postive trends and negative trends (see above) are plotted separately separately.

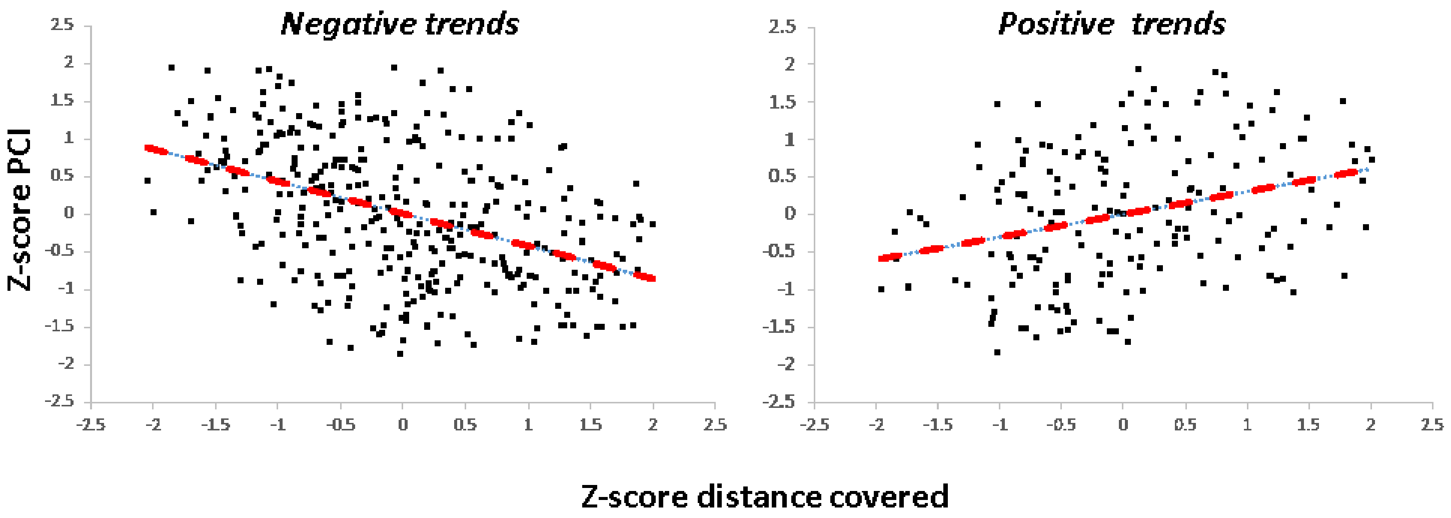

Figure 1-s: Left Panel: Z-scores of PCI are plotted against Z-scores of distance covered based on 61 data sets of 6 points each, lumped together from all individual negative trends showing better coordination (lower PCI values) with the higher gait speeds. **Right panel:** Z-scores of PCI are plotted against Z-scores of distance covered based on 31 data sets of 6 points each, lumped together from all individual positive trends showing worse coordination (higher PCI values) with the higher gait speeds. Trend lines represent statically significant weak (r=-0.35 and r= 0.31 negative and positive trends, respectively; p<0.0001).

While six data points are not sufficient to statistically power correlation analyses transformation of the data allows combining the data in order to reveal intrinsic trends of association between gait parameters and gait speed, as we detail here from the PCI parameter.
